# Exploring the utility of artificial intelligence in identifying progression of prostate cancer during active surveillance: A systematic review

**DOI:** 10.1038/s41391-026-01107-6

**Published:** 2026-05-12

**Authors:** Jianliang Liu, Dixon T. S. Woon, Wafy Ahmad, Thomas P. Cundy, Thilakavathi Chengodu, Nanadakishor Desai, Marimuthu Palaniswami, Marlon Perera, Nathan Lawrentschuk

**Affiliations:** 1 EJ Whitten Prostate Cancer Research Centre, Epworth Healthcare, Melbourne, VIC Australia; 2 Department of Surgery, The Royal Melbourne Hospital, University of Melbourne, Melbourne, VIC Australia; 3 University of Melbourne, Department of Surgery, Melbourne, VIC Australia; 4 Sir Peter MacCallum Department of Oncology, The University of Melbourne, Melbourne, VIC Australia; 5 Discipline of Surgery, University of Adelaide, Adelaide, SA Australia; 6 Department of Electrical and Electronic Engineering, University of Melbourne, Melbourne, VIC Australia

**Keywords:** Prostate cancer, Translational research

## Abstract

**Background:**

There is a recent surge in interest in the application of artificial intelligence (AI) in prostate cancer (PCa). When evaluating PSMA PET scans, AI has shown promise in detecting intraprostatic cancer and metastatic disease. This systematic review aims to assess the ability of AI to detect or predict the progression of PCa during AS.

**Methods:**

This systematic review was registered on PROSPERO (ID CRD42024529354) and conducted according to the Preferred Reporting Items for Systematic Reviews and Meta-analyses (PRISMA) guidelines. A comprehensive literature search was performed on Medline, Embase, Web of Science, and IEEE Xplore. Only studies evaluating AI in AS-eligible patients were included.

**Results:**

After screening 842 articles, 12 studies were suitable for inclusion. The included studies comprised of 4622 AS patients of whom 1022 experienced progressions. Only three studies utilised developed their AI purely on clinicopathological variables. The area under curve (AUC) of these three AI ranged between 0.65 and 0.76, and the AI algorithm in one study outperformed traditional logistic regression. The integration of MRI parameters particularly the use of radiomics improves the ability of AI to predict progression as compared to clinicopathological variables alone. AI was also able to analyse serial MRI during AS and performs on par with the Prostate Cancer Radiological Estimation of Change in Sequential Evaluation (PRECISE) scoring system. The AUC of the AI algorithms which included MRI parameters ranged between 0.65 and 0.95. One of the limitations was the variability in study methodologies and inclusion criteria. The Prediction Model Risk of Bias Assessment Tool (PROBAST) was used and none of the studies had high risk of bias.

**Conclusion:**

AI shows promise in detecting PCa progression during AS. However, this systematic review highlights the need for larger prospective studies with external validation before AI can be integrated into the AS process.

## Introduction

Prostate cancer (PCa) is the second most frequently diagnosed cancer globally [1]. Close to 40% of newly diagnosed PCa are classified as international society of urological pathology grade group (ISUP GG) 1 disease [2]. This group of PCa are generally indolent in nature and active surveillance (AS) has become the preferred management strategy [3–5]. One of the goal of AS is to delay or even completely avoid unnecessary treatment and associated side effects [3–5]. However, approximately 28% of patients on AS develop histopathological disease progression [6]. Accurately identifying progression during AS is crucial as it may trigger the need for active treatment and ensure that oncological outcomes are not compromised [7]. The current method of detecting progression during AS involves close monitoring with serial prostate-specific antigen, clinical examination, serial magnetic resonance imaging (MRI), and repeated prostate biopsies [3–5]. Although the introduction of MRI has revolutionised the management of PCa, clinically significant PCa (csPCa) are still occasionally missed [8, 9].

Assessing changes on serial MRI can be challenging [10]. The Prostate Cancer Radiological Estimation of Change in Sequential Evaluation (PRECISE) scoring system was developed to help standardise assessment on serial MRI [11]. The PRECISE score uses a 5-point scale to evaluate the likelihood of radiologic tumour progression, with scores 1 or 2 indicating radiological regression, score of 3 indicated stable radiological findings, and score of 4 or 5 indicated radiological progression. However, the PRECISE score was mainly validated in expert centres, there is a lack of consensus regarding the optimal method of measuring tumour size on MRI, and interpretation of prostate MRI is still reader experience dependent [10, 12, 13].

There has been a recent surge in interest in the application of artificial intelligence (AI) in medicine [14]. By harnessing its ability to process large amounts of data and incorporating radiomics features that may be imperceptible to the naked eye, AI can make complex decisions and improve predictive accuracy [15, 16]. This systematic review aims to assess the current ability of AI in detecting progression of PCa during active surveillance of PCa.

## Materials and methods

### Literature search strategy

This systematic review was registered on PROSPERO (International prospective register of systematic reviews) under the ID CRD42024529354. The Preferred Reporting Items for Systematic Reviews and Meta-analyses (PRISMA) guidelines was used. A comprehensive literature search was performed on Medline, Embase, Web of Science, and Institute of Electrical and Electronics Engineers (IEEE) Xplore. Key search terms used include artificial Intelligence, active surveillance, and prostate neoplasm.

### Eligibility criteria

The population, intervention, comparator, outcome (PICO) framework was used to guide our eligibility criteria. The study population included patients who were suitable for active surveillance as recommended by the NCCN (National Comprehensive Cancer Network), EAU (European Association of Urology), and AUA (American Urological Association) [3–5]. The decision-making process for AS is complex but the inclusion criteria broadly encompass low-risk and selected intermediate-risk PCa. Low-risk PCa was defined as clinical tumour stage (cT) ≤ 1-T2a (i.e tumour involving one half of prostate lobe or less), with PSA < 10 ng/mL, and ISUP GG 1 [17]. Intermediate-risk PCa which may be suitable for AS include ISUP GG 2 disease with <10% Gleason pattern 4 disease, PSA < 10 ng/mL, ≤cT2a, and low extent of tumour in biopsies ( ≤ 3 biopsy cores with ISUP GG 2 disease and ≤50% cancer involvement). Studies were excluded if patients had benign prostate histology or ISUP ≥ 3 disease.

The intervention in question is the application of AI to assess either clinical, pathological, or radiological variables during AS. The AI models were grouped into three classes: machine learning (ML), deep learning (DL), neural network (NN). ML is a broad field of AI where the algorithm processes data to make educated predictions [18]. DL is a subset of ML which uses complex algorithm with multiple decision-making layers [18]. NN is also a subset of ML which mimics the brain and uses interconnected units to process information [18]. The comparator may be between AI models, against traditional statistical analysis, or against the PRECISE scoring system [11]. The control will be patients who did not develop pathological or radiological progression during AS. Pathological progression was defined as increase in ISUP GG or Gleason score during repeat biopsy or subsequent radical prostatectomy (RP). Radiological progression was defined as new change on serial MRI that is concerning for progression of disease (such as PRECISE score of 4 or 5). The primary endpoint was to evaluate AI’s capability to detect or predict the progression of PCa during AS. This could be measured either as area under the curve (AUC), F1 scores or concordance index (C-index).

All English language original articles published from inception to November 2023 were considered. The following types of studies were excluded: case reports, studies evaluating less than five cases, reviews, commentaries, and conference proceedings.

### Screening and study selection

Titles, abstracts, and full-text screening were performed independently by two authors (J.L and W.A) and any unresolved conflicts were resolved by the senior author. The only automation tool used was Covidence (Veritas Health Innovation, Melbourne, VIC, Australia) to assist in the screening process and removal of duplicate articles. Data was manually extracted on to Excel 2013 (Microsoft Corporation, Redmond, CA, USA). Independent checks of the data were performed by the co-authors. Missing data were reported in the results.

### Risk of bias and STREAM-URO assessment

To assess the risk of bias and applicability of diagnostic and prognostic prediction model studies, the Prediction Model Risk of Bias Assessment Tool (PROBAST) was used [19]. Both the STREAM-URO and PROBAST checklists were independently completed by two authors (J.L and W.A), with any unresolved disagreements resolved by the senior author. The quality of each article was evaluated using the 26-item checklist from the standardised reporting of machine learning applications in urology (STREAM-URO) [20]. This framework was developed to ensure quality of study, improve reproducibility and interpretation of results, and to promote engagement and understanding of ML within the urological community.

## Results

### Screening process

**Fig. 1:**
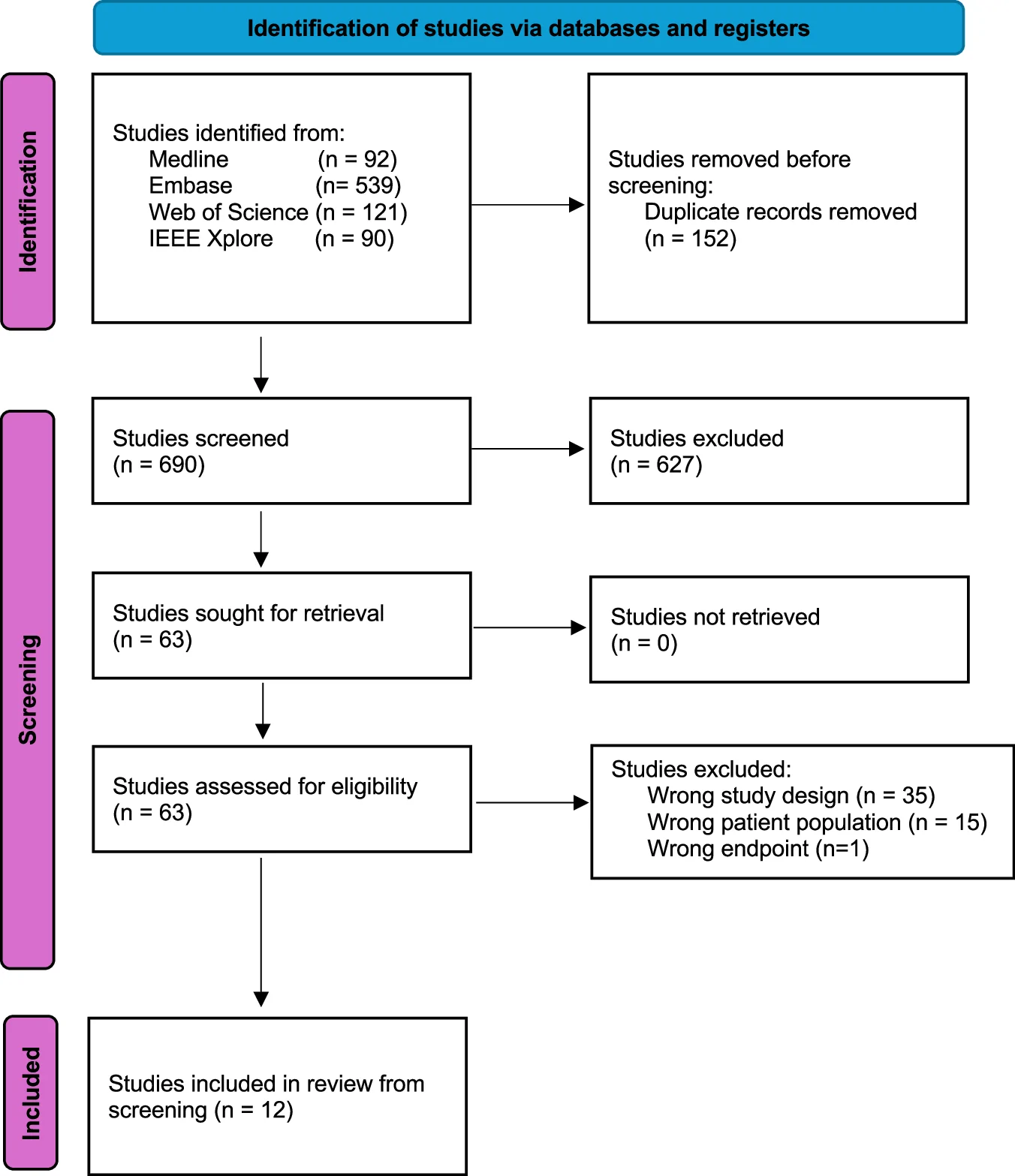
PRISMA flow diagram.

The search yielded 842 articles, of which 152 were duplicates (see Fig. 1). After excluding 627 articles during title and abstract screening, 63 studies remained for full text review. During full text review, 50 studies were excluded due to wrong study design (*n* = 35), inclusion of wrong patient population (*n* = 15), and wrong endpoint (*n* = 1). The remaining 12 studies were included in this systematic review.

### Characteristics of included studies and patients

The majority of the included studies were single-centre [21–30], only three studies were multicentre [31, 32] (See Table 1). There was only one prospective study [31], the remaining were retrospective in nature [21–30, 32]. The broad categories of AI techniques employed were ML [21], DL [30, 32], and NN [22, 26, 29, 31]. Five of the remaining studies compared between various types of AI algorithms [23–25, 27, 28].

**Table 1:** Characteristics and results of included studies.

Author and year	Study objective	AI model and study design	Inclusion/Exclusion criteria	Sample size	Data input	Findings	Strengths	Limitations
Chandramouli et al. 2020 [21]	Predict progression using quantitative histomorphometric features from diagnostic biopsy	ML Retrospective Single-centre	**Inclusion** cT1c, PSA ≤ 10 ng/mL, GS ≤ 7, ≤ 2 positive cores, ≤ 50% core involvement, PSA density ≤ 0.15 ng/mL and life expectancy ≤ 20 years	191 patients	initial H&E biopsy images	A combination of nuclear spatial arrangement, shape, and disorder features were associated with progression. DL had AUC of 0.64, sensitivity of 0.14, and specificity of 0.96	Stringent inclusion criteria	Small sample size
Sushentsev et al. 2023 [22]	Predict progression using PSA-based parameters	RNN Retrospective Single-centre	**Inclusion** Patients aged 50-80 years old enroled in AS, ISUP GG 1, PSA ≤ 20 ng/ml ISUP GG 2 with ≤10% pattern 4 **Exclusion** < 2years follow up. Previous treatment for benign disease	4508 serial PSA from 332 patients	Serial PSA, PSAD (adaptative and non-adaptive)	Without taking into account prostate size, non-adaptive PSAD demonstrated superior performance (AUC of 0.74) over both adaptive PSAD (AUC of 0.71) and PSA (AUC of 0.70) in predicting PCa progression	Median follow-up of 51 months	Risk of inter-reader variability of MRI prostate volume
Nayan et al. 2022 [23]	Predict progression on AS using clinicopathological variables	SVM vs. RF vs. ANN vs. ML-LR Retrospective Single-centre	**Inclusion** Very-low or low-risk PCa with ISUP GG 1, PSA < 10 ng/ml, cT ≤ 2a **Exclusion** No confirmatory biopsy	790 patients	Clinicopathological variables	In descending order, the F1 scores were as follows: SVM (0.59), ML-LR (0.52), ANN (0.39), RF (0.38), and T-LR (0.18). All ML models outperformed traditional LR (p < 0.001)	Large sample size with median follow-up of 6.29 years	Does not incorporate MRI
ElKarami et al. 2022 [24]	Predict progression of disease on MRI guided confirmatory prostate biopsy	NaiveBayes vs. SVM vs. RF vs. Bagging, vs. Ada- Boost Retrospective Single-centre	**Inclusion** Patients on AS who underwent MRI guided confirmatory prostate biopsy	592 patients	Clinicopathological variables	AdaBoost model was highly predictive of upgrading to ISUP GG 2 (AUC of 0.95). RF performed better at predicting upgrading to ISUP GG 3 (AUC 0.95).	Provided GINI-index overcome “black-box” of AI	Incomplete data
Sushentsev et al. 2021 [25]	Predict progression on AS by adding MRI radiomics to clinical parameters	KNN vs. GLMnet vs. SVM vs. RF Retrospective Single-centre	**Inclusion** patients with biopsy- proven PCa visible on both T2WI and ADC. ≥ 2 years follow up. ≥ 2 consecutive 3 T MRI scans performed on the same magnet. **Exclusion** No MR-visible lesions. Previous treatment for benign prostatic disease. Presence of hip or pelvic metal prosthesis	73 MR-visible lesions from 71 patients	Clinicopathological variables and MRI radiomics	Combining T2WI-derived radiomic features to clinical variables improved the AUC for predicting PCa progression from 0.61 to 0.75	Case control study	Small sample size
Oerther et al. 2023 [26]	Predict progression on MRI	CNN Retrospective Single-centre	**Inclusion** Patients who underwent mpMRI during AS **Exclusion** MRI performed as part of first guided biopsy before AS	83 MR-target biopsies of 56 patients	MRI	Detected ISUP GG ≥ 2 PCa with sensitivity of 96.4% and specificity of 25%	Stringent inclusion criteria	Small sample size
Arif et al. 2020 [31]	Develop an automatic method for identification and segmentation csPCa	CNN prospective Multicentre	**Inclusion** ISUP GG 1 PCa who were eligible for AS **Exclusion** Artefacts on MRI. Refused prostate biopsy.	292 patients	MRI	The CNN identified csPCa with an AUC ranging 0.65-0.89, sensitivity ranging 82-92%, and specificity ranging 43-76%	All MRI reviewed by single experience uroradiologist	Only validated in ISUP GG 1 patients
Xie et al. 2021 [27]	Predict upgrading in ISUP GG at RP in patients suitable for AS	NNA vs. SVM vs. RF vs. decision-tree Retrospective Single-centre	**Inclusion** Biopsy proven ISUP GG 1 or 2 who underwent MRI and RP **Exclusion** Previous treatment for PCa. Poor MRI quality. No visible lesion on MRI	59 patients	MRI	Ascending AUC of AI on testing were NNA (0.72), SVM (0.62), DT (0.67), and RF (0.63)	Stringent inclusion criteria	Small sample size
Sushentsev et al. 2022 [28]	Predict progression during AS using MRI delta-radiomics	PN vs. RF Retrospective Single-centre	**Inclusion** ≥ 2 years follow up. First and last 3 T MRI scans performed on the same magnet. ≥ 1 repeat targeted biopsy performed within a year of the last MRI **Exclusion** No MR-visible lesions. Previous treatment for benign prostatic disease. Presence of hip or pelvic metal prosthesis	64 patients	MRI delta-radiomics	The AUC for PRECISE (0.84) was slightly higher than the AUCs for PN (0.82), LASSO regression (0.78), and RF (0.81), but the differences were not statistically significant (p = 0.64, 0.43, and 0.57, respectively).	Compared between various type of AI and conventional analysis	Small sample size
Sushentsev et al. 2023 [29]	Predict progression during AS using MRI time series radiomics	RNN Retrospective Single-centre	**Inclusion** ISUP GG 1 or ISUP GG 2 with ≤ 10% pattern 4, ≥ 2 years follow up, ≥ 1 MRI visible lesion, ≥ three 3 T MRI Repeat targeted biopsy within 12 months of final MRI **Exclusion** Previous treatment for benign prostatic disease. Presence of hip or pelvic metal prosthesis	297 scans from 76 patients	PSA, PSAD, MRI time series radiomics, MRI delta-radiomics	Long short-term memory RNN combined with time series radiomics and serial PSA density achieved AUC of 0.86, comparable to PRECISE scoring system (AUC of 0.84)	Stringent inclusion criteria and compared between AI developed using various data input	Small sample size
Roest et al. 2022 [32]	Develop a DL for the longitudinal analysis of consecutive MRI to detect sPCa	DL Retrospective Multicentre	**Inclusion** Patients on AS that underwent MRI and prostate biopsy. **Exclusion** Has csPCa prior to biopsy	1513 patients of whom 78 had serial MRI	Clinical and MRI variables	Combining clinical variables with serial MRI improved DL’s diagnostic performance from AUC of 0.81 to 0.86. The AI model outperformed radiologists (AUC of 0.69)	Longitudinal analysis	Mixture of 3 T and 1.5 T scanners
Lee et al. 2022 [30]	Prediction of risk of progression during AS	DDHL Retrospective Single-centre	**Inclusion** Low to favourable intermediate risk PCa **Exclusion** Insufficient information. < 1 year follow up	585 patients	Clinicopathological and MRI variables	With 3 years of data collection and 3 years follow-up, the DDHL model (C-index 0.79), outperformed Cox regression (C-index 0.7)	Compared against traditional methods of predicting risk of progression	Incomplete data regarding MRI

*AS* Active surveillance, *ADC* Apparent diffusion coefficient, *AUC* Area under the curve, *ANN* Artificial neural network, *PCa* Clinically significant prostate cancer, *cT* Clinical tumour stage, *CAD* Computer-assisted diagnosis system, *CNN* Convolutional neural network, *DL* Deep learning, *DDHL* Dynamic-DeepHit-Lite, *GLMnet* General linear model, *GS* Gleason sum, *H&E* Hematoxylin and eosin, *ISUP GG* International Society of Urogenital Pathology grade groups, *KNN* K-nearest neighbours, *LASSO* Least absolute shrinkage and selection operator, *LR* Logistic regression, *MRI* Magnetic resonance imaging, *NNA* Nearest neighbour algorithm, *PN* Parenclitic networks, *PRECISE* Prostate Cancer Radiologic Estimation of Change in Sequential Evaluation, *PSAD* Prostate-specific antigen density, *RP* Radical prostatectomy, *RF* Random forests, *RNN* Recurrent neural network, *ROI* Region of interest, *SVM* Support vector machine, *T2WI* T2-weighted Imaging.

Across the 13 studies, a total of 4622 patients were included, of whom 1022 experienced pathological progression (See Table 1). The median age of patients ranged between 63 and 71 years old, and the median PSA ranged between 4.3 and 13 ng/ml. The majority of studies included low-risk and selected intermediate-risk PCa suitable for AS [21, 22, 25, 27–30]. However, three studies only included ISUP GG 1 PCa [23, 26, 31], and the criteria for AS were not clearly defined in two studies [24, 32]. Patient characteristics were not reported in one study [21]. Additionally, four of the included articles originated from Sushentsev et al., which may result in some overlapping of included patients [22, 25, 28, 29]. Median follow-up was not reported in four studies[21, 26, 27, 31]; in the remaining studies, the median follow-up ranged between 21 and 75.5 months.

### Risk of bias assessment and STEAM-URO grading of included studies

**Fig. 2:**
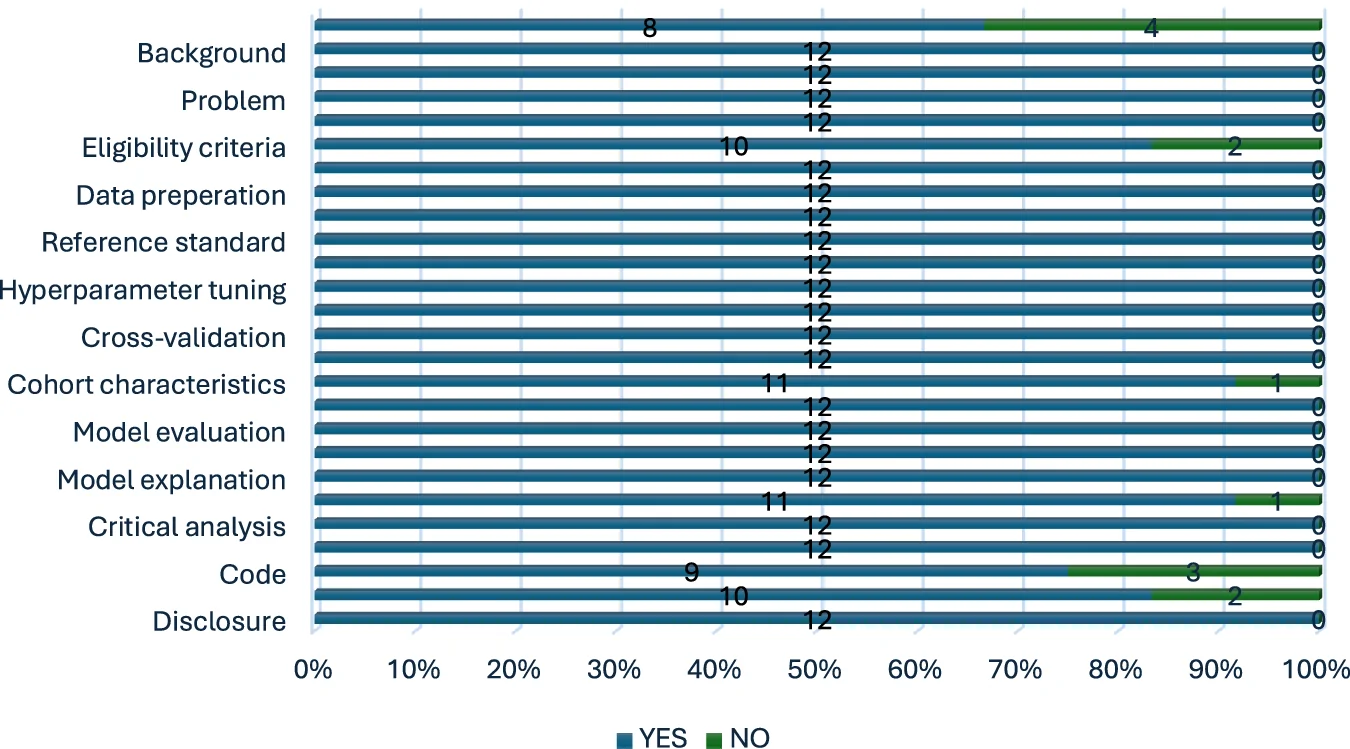
Grading of the included studies. This figure outlines standardised reporting of machine learning applications in urology (STREAM-URO) grading of the included studies.

During the PROBAST assessment (See Table 2), two studies had an unclear risk of bias due to unclear inclusion criteria for AS [24, 32]. Additionally, missing data were replaced with average values in one of the studies [24]. However, none of the included studies had a high risk of bias. Concerns regarding applicability were high in four studies because they only included patients with prostatic lesions visible on MRI [25, 27–29]. During STEAM-URO grading (see Fig. 2), the mean score was 24 out of 26. The main areas where studies lost points were not mentioning the use of AI in the title (*n* = 4) [21, 25, 28, 29], and not describing the eligibility criteria for the inclusion of patients (*n* = 2) [24, 32].

**Table 2:** The prediction model risk of bias assessment tool (PROBAST) of included studies.

Study	ROB				Applicability concerns			Overall	
	Participants	Predictors	Outcome	Analysis	Participants	Predictors	Outcome	ROB	Applicability
Chandramouli et al. 2020 [21]	Low	Low	Low	Low	Low	Low	Low	Low	Low
Sushentsev et al. 2023 [22]	Low	Low	Low	Low	Low	Low	Low	Low	Low
Nayan et al. 2022 [23]	Low	Low	Low	Low	Unclear	Low	Low	Low	Unclear
ElKarami et al. 2022 [24]	Unclear	Low	Low	Unclear	Low	Low	Low	Unclear	Low
Sushentsev et al. 2021 [25]	Low	Low	Low	Low	High	Low	Low	Low	High
Oerther et al. 2023 [26]	Low	Low	Low	Low	Unclear	Low	Low	Low	Unclear
Arif et al. 2020 [31]	Low	Low	Low	Low	Unclear	Low	Low	Low	Unclear
Xie et al. 2021 [27]	Low	Low	Low	Low	High	Low	Low	Low	High
Sushentsev et al. 2022 [28]	Low	Low	Low	Low	High	Low	Low	Low	High
Sushentsev et al. 2023 [29]	Low	Low	Low	Low	High	Low	Low	Low	High
Roest et al. 2022 [32]	Unclear	Low	Low	Low	Unclear	Low	Low	Unclear	Unclear
Lee et al. 2022 [30]	Low	Low	Low	Low	Low	Low	Low	Low	Low

*ROB* Risk of bias.

### Predicting progression during active surveillance using clinicopathological variables

In total there was only one study which exclusively trained their AI algorithm with pathological variables and another two which trained their AI models with clinicopathological variables. Chandramouli et al. [21] employed quantitative histomorphometric features which are computationally derived descriptors of tissue morphology extracted from initial hematoxylin and eosin biopsy slides. A DL algorithm utilising the DenseNet architecture was trained and compared to the quantitative histomorphometric features. The integration of the top six quantitative histomorphometric features allowed for accurate identification of patients who were at risk of pathological progression (AUC of 0.75). This model outperformed the average predictive performance of conventional nomograms (AUC of 0.59).

Only one study explored the utility of various PSA parameters in predicting progression of PCa during AS (See Table 1). When using the baseline prostate gland size from initial MRI to calculate PSA density (PSAD), it is described as non-adaptative PSAD (PSAD_NA_). Whereas the use of new prostate gland size from any new MRI performed is described as adaptive PSAD (PSAD_A_). Sushentsev et al. [22] employed a long short-term memory recurrent NN (RNN) to determine which of these PSA parameters best predicts pathological and/or radiological progression during AS. PSAD_NA_ (AUC of 0.75 to 0.76) appears to outperform PSAD_A_ (AUC of 0.70 to 0.74) and PSA (AUC of 0.68 to 0.70), particularly in patients with prostate size <55 ml. However, in patients with prostate size >55 ml, the long short-term memory recurrent NN appears to perform the best with PSA (AUC of 0.73) as compared to PSAD_NA_ (AUC of 0.68) and PSAD_A_ (AUC of 0.65). This finding was hypothesised to be due to PSAD_NA_ tendency to overestimate the true PSAD in patients with a prostate size < 55 ml, while in patients with a prostate size > 55 ml, a larger change in PSA is needed to alter PSAD values.

Another study utilised clinicopathological variables such as patient’s age, family history, cT stage, PSA level, prostate size on transrectal ultrasound, and prostate biopsy results (See Table 1). Nayan et al. [23] found that support vector machine (SVM) which is a subtype of ML performed the best at predicting pathological progression during AS when compared to ML logistic regression (ML-LR), artificial NN (ANN), and random forest (RF) (F1 score of 0.586, 0.522, 0.392, 0.376 respectively). Additionally, all these AI algorithms outperformed traditional logistic regression (F1 score of 0.18, *P* < 0.001). It is worth noting this study only included ISUP GG 1 patients.

### Predicting progression during active surveillance using single time point MRI

ElKarami et al. [24] developed and compared between various ML algorithms including SVM, NaiveBayes, RF, Bagging, and AdaBoost. The endpoint was to predict pathological progression on MRI target biopsy. However, the radiological findings from the MRI itself were not incorporated into the AI algorithms. ElKarami et al. [24] found that AdaBoost model performed the best at predicting pathological progression to ISUP GG 2 disease (AUC of 0.952) and that RF performed the best when predicting pathological progression to ISUP 3 disease (AUC of 0.947).

Sushentsev et al. [25] extracted intratumoural radiomic features from T2-weighted imaging (T2WI) and apparent diffusion coefficient (ADC) of MRI. Various combination of PSA, PSAD, and radiomics were then used to develop and compared between different ML algorithms such as k-nearest neighbours (KNN), linear discrimination analysis (LDA), general linear model (GLMnet), SVM, LR, and RF. Sushentsev et al. [25] demonstrated that KNN performed the best at predicting pathological and radiological progression. When T2WI radiomics was added to clinical parameters, the AUC improved from 0.61 to 0.75.

Two of the included studies developed AI algorithms to predict pathological progression in patients with ISUP GG 1 PCa. Oerther et al. [26] utilised a convolutional NN (CNN) previously developed for automatic lesion detection, segmentation, and classification. This CNN was applied to patients on AS who underwent multiparametric MRI and was able to detect ISUP GG ≥ 2 PCa with a sensitivity of 96.4% and specificity of 25%. Arif et al. [31] incorporated findings from multiparametric MRI to develop a CNN that could automatically identify and segment csPCa (ISUP GG ≥ 2). For lesions > 0.03 cc to 0.5 cc, the CNN demonstrated an AUC between 0.65 and 0.89, sensitivity between 82% and 92% and specificity of 43% to 76%.

Xie et al. [27] sought to create a ML model for predicting pathological progression from prostate biopsy to RP in patients suited for AS. They extracted ninety-four texture features from the ADC of MRI, identifying six features (four histogram features, one absolute gradient feature, and one GLCM feature) as statistically significant for distinguishing between patients with and without pathological upgrading (*p* < 0.05). These features did not differ significantly between patients with ISUP GG 1 and ISUP GG 2 on final RP histopathology. These six features were than used to train various ML algorithms. The nearest neighbour algorithm (NNA) performed the best with an AUC of 0.872, followed by the SVM with an AUC of 0.861, and then the RF model with an AUC of 0.728.

### Predicting progression during active surveillance using longitudinal analysis of serial MRI

Sushentsev et al. [28] developed an AI algorithm which can predict pathological progression on serial MRI. To account for the changes over time, arithmetic difference between the final and baseline MRI features were computed into delta-radiomics features. These T2WI- and ADC-derived delta-radiomics were then used to develop parenclitic networks (PN), least absolute shrinkage and selection operator (LASSO) LR, and RF models. There was no statistical difference between the AUC of all three models. Although not statistically significant, the AUC of PRECISE score (0.844) was higher when compared to PN (0.815), LASSO LR (0.78), and RF (0.809). However, RF (0.926) achieved the highest sensitivity for predicting pathological progression when compared to PRECISE score (0.741), PN (0.852), and LASSO LR (0.704). Similarly, RF (0.926) had the highest negative predicting value for predicting pathological progression when compared to PRECISE score (0.837), PN (0.875), and LASSO LR (0.800).

In a separate study, Sushentsev et al. [29] incorporated time-series radiomics to allow for longitudinal analysis of the patient. This involves extracting quantitative features from MRI over a sequence of time points. Sushentsev et al. [29] demonstrated that a RNN combining time-series radiomics with serial PSA density (AUC of 0.86) significantly outperformed a model combining delta-radiomics and delta-PSA density (AUC of 0.75; *p* = 0.048). The RNN appears to be comparable to the PRECISE scoring system (AUC of 0.84; *p* = 0.710).

Roest et al. [32] developed a DL for the longitudinal analysis of consecutive biparametric MRI in order to detect csPCa (ISUP GG > 1). The AI model that incorporated serial MRI achieved greater diagnostic accuracy (AUC of 0.81) than the model using only a single MRI (AUC 0.73; *p* = 0.04). When clinical variables (age, PSA, and PSAD) were added, the diagnostic accuracy further increased (AUC of 0.86). Overall, the AI model outperformed radiologists (AUC of 0.69; *p* = 0.02). Lee et al. [30] developed a DL-based individualised longitudinal survival model using Dynamic-DeepHit-Lite (DDHL) using clinicopathological and MRI variables. Progression was more broadly defined by Lee et al. [30] as increase in ISUP GG disease, or increase PSA ≥ 20 ng/mL, or increase in tumour staging. When three years of follow-up data was incorporated, the DDHL model (C-index of 0.79) outperformed Cox regression analysis (C-index of 0.67) and landmarking method (C-index of 0.7).

## Discussion

There has been an increased uptake in AS for managing low-risk prostate cancer, driven by a desire to avoid overtreatment and its associated side effects [33]. Accurately identifying patients who have disease progression during AS is crucial, as it allows for timely intervention with active treatment, ensuring better oncological outcomes. The findings in this systematic review suggest that trends in PSA-related parameters is one of the key clinicopathological factors for developing AI algorithms to predict progression during AS. The utility of MRI during AS was highlighted by its ability to improve the performance of these AI models, particularly when incorporated with PSA metrics [25]. AI algorithms trained solely on clinicopathological factors had AUCs ranging between 0.65 and 0.76, while those incorporating MRI parameters had AUCs ranging between 0.65 and 0.95.

Assessments of serial MRI can be challenging due to variability in interpretation. Hence, the PRECISE score was developed to standardise serial MRI reporting for AS patients. However, the PRECISE score has only been validated in expert centres, limiting its widespread applicability [13]. This systematic review found that AI algorithms ability to assess serial MRI appears comparable to the performance of the PRECISE score [28, 29]. AI algorithms could potentially help further minimise inter-reader variability particularly in centres less experienced with the PRECISE score. Furthermore, AI can increase diagnostic efficiency by providing a preliminary analysis before a radiologist’s review. Another advantage of AI over traditional methods like the PRECISE score is its ability to incorporate radiomics, which allows for a more nuanced analysis of imaging data. For example, models that combine PSA metrics with MRI-derived radiomic features have shown enhanced accuracy in identifying patients likely to experience progression [25]. This indicates that AI can effectively integrate multimodal data for a more comprehensive assessment of disease dynamics.

The current risk stratification for AS primarily relies on the D’Amico classification, which was developed in 1998 and mainly validated for predicting biochemical recurrence after curative treatment [34]. Moreover, these classification tools do not take into account changes in serial MRI. There is a need to improve our patient selection in order to reduce the current estimated 28% risk of histopathological disease progression during AS [6, 35]. The study by Chandramouli et al. [21] showed that AI could evaluate prostate biopsies and identify quantitative histomorphometric features that accurately predict patients at risk of pathological progression during AS. This model even outperformed the average predictive performance of conventional nomograms, suggesting that AI could play a crucial role in refining patient selection for AS in the future. Particularly as we move towards a more personalised approach to prostate cancer management.

We acknowledge several limitations of this systematic review. There was substantial variability in the design and methodology of the included studies, including the lack of standardisation in the eligibility criteria for AS. This made direct comparison between studies challenging and hindered the conduct of a meta-analysis. Most studies were retrospective, single-centred, and lack of external validation which limits the generalisability of their results. Furthermore, endpoints and methods of assessment were not consistent. For example, majority of studies determined pathological progression by comparing initial prostate biopsies to subsequent biopsies, but one study used subsequent RP histopathology as comparison. It is recognised that there is a tendency for upstaging from prostate biopsy to RP histopathology [36]. Future studies should standardise endpoints, such as defining pathological progression as an increase in Gleason score or ISUP Grade Group in subsequent repeat prostate biopsies.

Applying AI to the AS process has its limitations, including privacy concerns and the challenge of the “black box” nature of many AI models, which can make their decision-making processes opaque and difficult to interpret [37]. Additionally, the variability in AI model performance due to differences in data quality, sample size, and population diversity across studies limits its clinical application.

Although the current state of AI appears promising for helping us identify patients at risk for progression during AS, it is not ready for widespread clinical application. Larger, prospective, multicentre studies are needed to train, validate, and test AI algorithms before integrating them into clinical practice. Another area of interest is whether AI could help translate information into a more patient-oriented format. This is important to improve patient’s understanding of their medical condition and help with informed consent. This is mainly done through natural language processing (NLP) models, which are AI algorithms designed to understand and interpret human language. While a useful tool for patient education, existing NLP carries the risk of providing inaccurate information [38–40]. The integration of NLP into AI algorithms designed for predicting risk of progression during AS could be the next step in facilitating communication with patients. Additionally, this can support shared decision-making between patients and clinicians by providing personalised and easy to understand explanations of risk of progression.

Future studies could also explore several other areas, such as the utility of AI prior to confirmatory biopsy. An area of progression that has not been explored in the included studies is the increase in volume of PCa on repeat biopsy [7]. Future AI tools could potentially help us differentiate which high-volume ISUP Grade Group 1 PCa cases may benefit from active treatment rather than AS. There is also growing interest in using PSMA PET scans in AS to identify clinically significant prostate cancer (csPCa) [41, 42]. Further research is needed to evaluate this, but it could become another variable that can be used to train AI algorithms in predicting which patients are likely to progress during AS.

## Conclusion

In conclusion, AI shows promise in identifying patients who are likely to progress during AS. During longitudinal assessment, AI performs comparably to the PRECISE score. However, further studies with large, multi-centre, prospective data are needed to refine these models and validate these findings before they can be incorporated into clinical practice.
